# Engineering enhanced cellobiohydrolase activity

**DOI:** 10.1038/s41467-018-03501-8

**Published:** 2018-03-22

**Authors:** Larry E. Taylor, Brandon C. Knott, John O. Baker, P. Markus Alahuhta, Sarah E. Hobdey, Jeffrey G. Linger, Vladimir V. Lunin, Antonella Amore, Venkataramanan Subramanian, Kara Podkaminer, Qi Xu, Todd A. VanderWall, Logan A. Schuster, Yogesh B. Chaudhari, William S. Adney, Michael F. Crowley, Michael E. Himmel, Stephen R. Decker, Gregg T. Beckham

**Affiliations:** 1 Biosciences Center, National Renewable Energy Laboratory, Golden, CO 80401 USA; 2 National Bioenergy Center, National Renewable Energy Laboratory, Golden, CO 80401 USA; 3 Life Sciences Division, Institute of Advanced Study in Science and Technology (IASST), Guwahati, India

## Abstract

Glycoside Hydrolase Family 7 cellobiohydrolases (GH7 CBHs) catalyze cellulose depolymerization in cellulolytic eukaryotes, making them key discovery and engineering targets. However, there remains a lack of robust structure–activity relationships for these industrially important cellulases. Here, we compare CBHs from *Trichoderma reesei* (*Tr*Cel7A) and *Penicillium funiculosum* (*Pf*Cel7A), which exhibit a multi-modular architecture consisting of catalytic domain (CD), carbohydrate-binding module, and linker. We show that *Pf*Cel7A exhibits 60% greater performance on biomass than *Tr*Cel7A. To understand the contribution of each domain to this improvement, we measure enzymatic activity for a library of CBH chimeras with swapped subdomains, demonstrating that the enhancement is mainly caused by *Pf*Cel7A CD. We solve the crystal structure of *Pf*Cel7A CD and use this information to create a second library of *Tr*Cel7A CD mutants, identifying a *Tr*Cel7A double mutant with near-equivalent activity to wild-type *Pf*Cel7A. Overall, these results reveal CBH regions that enable targeted activity improvements.

## Abstract (web summary)

Cellobiohydrolases (CBHs) are critical for natural and industrial biomass degradation but their structure–activity relationships are not fully understood. Here, the authors present the biochemical and structural characterization of two CBHs, identifying protein regions that confer enhanced CBH activity.

## Introduction

Plant cell walls are highly evolved heterogeneous composite structures, which are a significant challenge to deconstruct^1,2^. The recalcitrant polymers cellulose and hemicellulose comprise the majority of plant cell wall polysaccharides. To date, many processes have been developed to produce renewable fuels and chemicals from biomass-derived sugars, ranging from ethanol to higher alcohols via fermentation in genetically modified organisms^3^ to hydrocarbons produced biologically^4^ or catalytically^5^. Accordingly, there is significant impetus to develop cost-effective sugar production methods for upgrading to fuels and chemicals. Most current leading options to produce sugars from lignocellulose utilize a thermochemical pretreatment step that renders the plant cell wall more amenable to the effective application of enzyme cocktails in a second step, which deconstructs cellulose and hemicellulose to soluble sugars^6^. The enzymatic hydrolysis step alone represents a significant fraction (up to 25%) of the operating and capital cost of lignocellulosic biofuel production^7^, and towards this, significant efforts have been focused on development of enhanced industrial enzymes^8,9^.

**Fig. 1:**
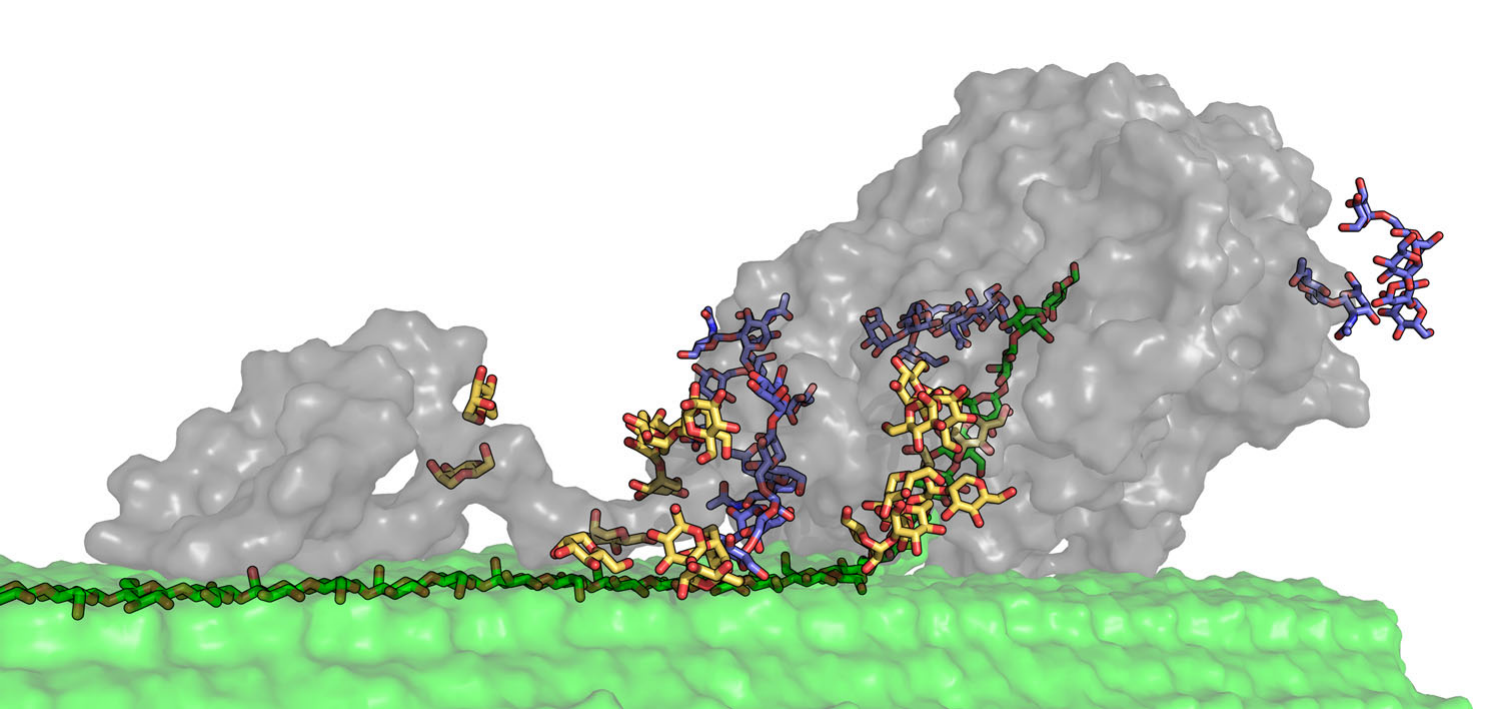
Multi-modular structure of Family 7 cellobiohydrolases. The GH Family 7 CBH from *T. reesei* is shown in the catalytically active complex on a cellulose microfibril. Shown in gray are the enzyme domains: at right is the catalytic domain (CD), at left is the carbohydrate-binding module (CBM), and connecting the two is the linker domain bound to the cellulose surface. Structure adapted from Zhong et al.^76^ The cellulose microfibril is shown in green ‘surface’ representation; ‘sticks’ are also shown for the strand upon which Cel7A is complexed. *O*-glycans are shown on the linker and CBM in yellow; *N-*glycans are shown in dark blue on the catalytic domain

Many biomass-degrading enzymes under development today are based on fungal cellulase secretomes. The emphasis on fungal cocktails originated from the isolation of the fungus *Trichoderma reesei* in the late 1940s, which has grown into an important platform for the production of cellulases at extremely high protein titers^8^. In most eukaryotic cellulase systems^10^, and especially in cellulolytic filamentous fungi, Glycoside Hydrolase Family 7 (GH7) cellobiohydrolases (CBHs) are often the main enzymes produced in natural secretomes^8^, likely because these enzymes provide the majority of the hydrolytic activity for cellulose conversion to glucose. GH7 cellulases are particularly important to industrial fungal cellulase cocktails, as the current lignocellulosic biorefineries operating worldwide predominantly use fungal-based cellulase systems. GH7 CBHs have therefore been the focus of many structural and biochemical studies and primary targets for cellulase engineering^8,11–22^. To date, many GH7 CBH structures have been reported including the well-studied *T. reesei* Cel7A, denoted *Tr*Cel7A. As with many GH7 CBHs, *Tr*Cel7A is a multi-modular enzyme with a Family 1 carbohydrate-binding module (CBM), which is responsible for binding to cellulose, connected to the catalytic domain (CD) by a flexible, glycosylated linker^8,11,12,23,24^, as illustrated in Fig. 1.

Rational engineering of CBHs requires insight into the structure and function of the individual domains, as well as the entire enzyme complexed with cellulose. Hypotheses as to the role of the individual domains have evolved since the first structural studies of multi-domain cellulases^11,12,24,25^. The Family 1 CBM is thought to be primarily responsible for increasing the binding affinity of a given cellulase CD to the cellulose substrate, thus ensuring a high catalyst concentration at the solid surface. Many protein engineering studies conducted with the CBM have highlighted the role of aromatic and polar residues on binding^26–28^ and more recently, the importance of glycosylation on binding and stability^29–31^. Similarly, the linker acts as a flexible tether between the CBM and CD^32^, and was demonstrated to aid cellulose binding^33^. The CD in GH7 CBHs exhibits a 50 Å-long tunnel wherein a single cellulose chain is threaded, complexed, and hydrolyzed to the disaccharide cellobiose. The putative catalytic cycle of Cel7A includes surface binding, location of a free cellulose chain end, chain complexation, hydrolysis, product expulsion, and processivity until the cellulase consumes an entire chain or becomes stuck due to obstacles in its path^34,35^.

Despite efforts to engineer GH7 CBHs for improved activity, relatively few successes have been reported. For direct improvement of specific activity, high-throughput screening approaches are limited because cellulose deconstruction requires many hours to days to reach relevant conversions. Moreover, for fungal enzymes, glycosylation is important for activity^36,37^, precluding many standard expression hosts, and native filamentous fungi are not yet typically amenable to high-throughput expression. Thus, nascent cellulase engineering efforts have been limited to screening relatively small sets of rationally guided or computationally designed mutations for higher thermal stability and subsequent gains in activity at higher conversion temperatures^18–20,38–43^. To date, there are few studies that demonstrate significant increases in activity on industrially relevant substrates above that reported for several well-studied GH7 CBHs. Indeed, genomics and metagenomics studies have elucidated a broad library of enzymes in many of the most important GH families for industrial biomass conversion, yet self-consistent activity comparisons amongst these are scarce^8^. This lack of information on GH7 CBHs represents a remaining gap in cellulase engineering that limits our collective ability to develop reliable structure–activity relationships for these important natural and industrial enzymes.

Towards the ultimate goal of developing more detailed structure–activity relationships in GH7 CBHs, here we report the detailed characterization of a multi-modular GH7 CBH from the fungus *Penicillium funiculosum*^44^, denoted *Pf*Cel7A. This enzyme exhibits a greater than 1.6× superiority in performance over the well-studied *Tr*Cel7A on a process relevant biomass substrate. To understand the reasons for this greater activity, we conduct domain swapping experiments with the CBM, linker, and CD from the two parent enzymes to create a library of *Tr*Cel7A and *Pf*Cel7A-based chimeras and compare their performance; these experiments suggest that the *Pf*Cel7A CD is the primary, but not the only, driver of the activity differences in the parent enzymes. From there, we solve the X-ray crystal structure of the *Pf*Cel7A CD, from which we design a second mutant library wherein eight changes are swapped individually into the *Tr*Cel7A parent enzyme. This reveals two mutations that, when combined, result in a *Tr*Cel7A-parent based mutant with performance on pretreated biomass approaching that of the *Pf*Cel7A parent, thus revealing two important motifs in GH7 CBHs that can result in dramatic performance differences. The functional importance of these two motifs is explored via molecular dynamics (MD) simulations of the enzymes both in solution and complexed on a cellulose microfibril.

## Results

### Characterization of CBH *Pf*Cel7A

The *Pf*Cel7A and *Tr*Cel7A enzymes were expressed in a recently reported *T. reesei* expression system^45^. Enzyme production and purification are described in the Methods. The Michaelis–Menten kinetics of both enzymes on the small molecule substrate *p*NPL, *T*_max_ (calorimetrically measured approximate midpoint of thermal denaturation), and pH and temperature activity optima are summarized in Supplementary Table 1 and Supplementary Fig. 1.

**Fig. 2:**
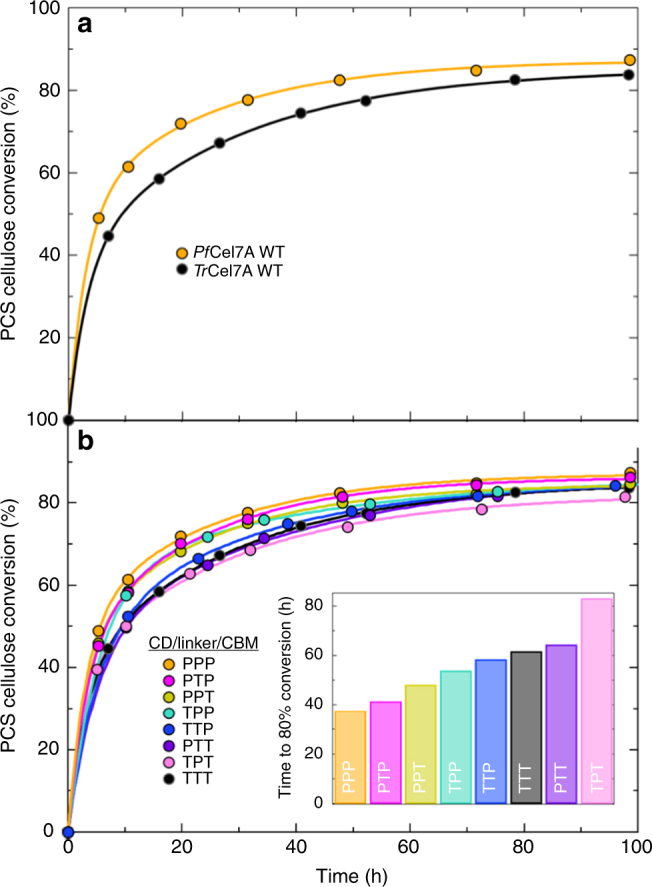
Activity data on dilute acid pre-treated corn stover. Glucan conversion is shown as a function of time on PCS for **a** wild-type *Pf*Cel7A and wild-type *Tr*Cel7A and **b** the domain swap chimera library. The P and T stand for *P. funiculosum* and *T. reesei*, respectively in the domain architecture in the order of CD, linker, and CBM. The lines represent double-exponential fits to the data. These assays were performed at *T* = 40 °C and pH = 5.0. The inset graph shows the time to 80% conversion (in hours) of the double exponential fit to each data trend. Graphs with these fits are available in Supplementary Fig. 2. Experiments were performed in triplicate; error bars represent the standard error of the mean (SEM) and are smaller than the data markers

Figure 2 shows the activity comparisons between *Tr*Cel7A and *Pf*Cel7A on dilute acid pretreated corn stover (PCS) at 40 °C. Although 40 °C is lower than the optimal temperature for these CBHs, enzymatic saccharification in the biorefinery typically employs the simultaneous saccharification and fermentation (SSF) approach. SSF operating temperature is limited by the fermentative organism and rarely has SSF operation achieved an operating temperature significantly above 40 °C^46^. CBH activity assays were performed in the presence of a highly active Family 5 endoglucanase (E1) from *Acidothermus cellulolyticus*^47^ and a β-glucosidase from *Aspergillus niger* for enzyme synergy and to alleviate product inhibition. We utilize this cocktail of three enzymes (rather than an isolated CBH) because (1) this is the context that CBHs are used industrially and (2) the rate-limiting step in the CBH processive cycle is different with and without accessory enzymes^48,49^. The total enzyme loading was 30.4 mg total enzyme per gram of cellulose with a mass ratio of 56:3.8:1 of the GH7 CBH:GH5 endoglucanase:β-glucosidase. Cellulase performance is measured as the time to reach 80% conversion (via a double exponential fit). For industrial purposes and techno-economic modeling, the time-to-target with 80% glucan conversion is the primary relevant metric used here to assess cellulase performance^7,50^. Fig. 2a shows that wild-type (WT) *Pf*Cel7A reaches 80% conversion more than 1.6× as fast (38 h relative to 62 h) compared to WT *Tr*Cel7A. (Also note that the conclusions reached in what follows are invariant to the choice of conversion target; if one chooses any conversion target between 50 and 80%, the activity superiority of *Pf*Cel7A over *Tr*Cel7A is always greater than a factor of 1.6; see Supplementary Table 2.)

The results from the screening are shown in Fig. 2b. Based on the time to reach 80% conversion, the performance of the *Pf*Cel7A parent is slightly reduced upon swapping either the linker or CBM for the corresponding *Tr*Cel7A domain (PTP and PPT, respectively). If both domains are swapped (PTT), however, performance is reduced to that of WT *Tr*Cel7A. Conversely, swapping the linker of the *Tr*Cel7A parent reduces the performance (TPT), whereas swapping the CBM or the CBM/linker for that of *Pf*Cel7a (TTP and TPP, respectively) improves *Tr*Cel7A performance. Given the lack of a completely uniform trend in terms of the effect of a single domain on activity, clearly there are interactions between domains that require further study^51^. Though there is only one outlier in our study (and the effect is minor), these results demonstrate a knowledge gap in the functional interconnectedness of the CBM, linker, and catalytic domain in conversion of cellulose to soluble sugars. In addition, this result suggests that significant performance improvements can be made to cellulases based on natural diversity screening and chimera library generation.

### Structural characterization

**Fig. 3:**
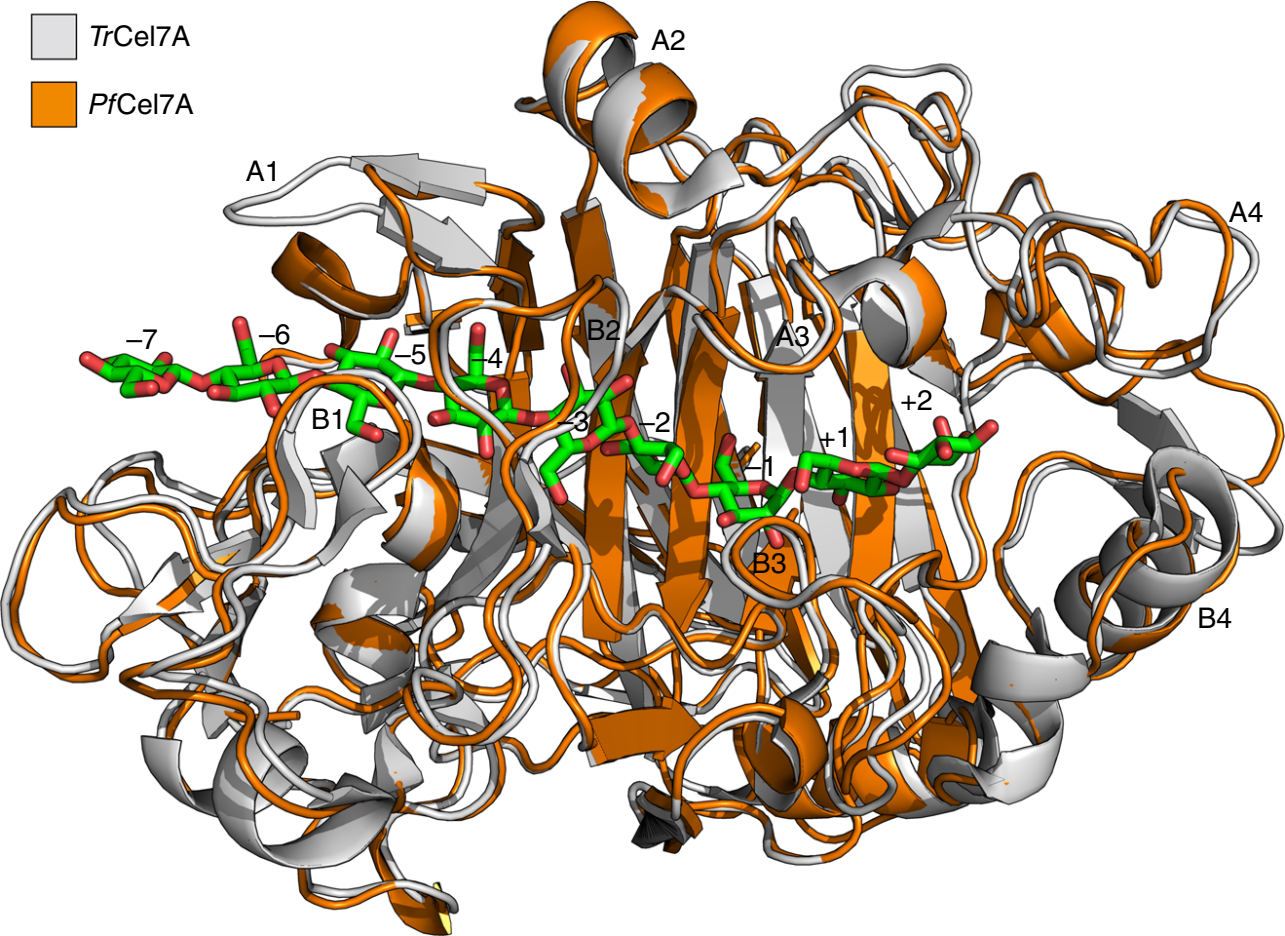
The structure of *Pf*Cel7A. The structure of *Pf*Cel7A (PDB code 4XEB), shown in orange, along with that of the canonical GH7 CBH *Tr*Cel7A (PDB code 4C4C) in light gray. The nine substrate binding sites (−7 to + 2) and the eight loops that form the substrate binding tunnel (A1, B1, etc.) are labeled according to the standard convention. The cellononaose ligand from the *Tr*Cel7A Michaelis complex (PDB code 4C4C) is shown in green ‘sticks’. For key areas of differences in these structures that inspired the construction of a library of CD mutants, see Supplementary Fig. 5

Motivated by the superior performance imparted by the *Pf*Cel7A CD relative to that of *Tr*Cel7A, we sought to understand the structural roots of this functional difference by solving the X-ray crystal structure of the *Pf*Cel7A CD (PDB code 4XEB) to 1.70 Å resolution (Table 1), which is shown in Fig. 3 aligned with *Tr*Cel7A (PDB code 4C4C)^17^. A stereo image of a portion of the electron density map is available in Supplementary Fig. 3.

**Table 1:** X-ray data collection and refinement statistics

	*Penicillium funiculosum* Cel7A
Data collection	
Space group	*C*2
Cell dimensions	
* a*, *b*, *c* (Å)	71.0, 61.8, 85.8
*α*, *β*, *γ* (°)	90.0, 98.8, 90.0
Resolution (Å)	50.0 1.7 (1.8–1.7)
* R*_merge_	0.103 (0.400)
* I*/*σI*	13.0 (1.7)
Completeness (%)	99.0 (94.4)
Redundancy	8.6 (2.1)
Refinement	
Resolution (Å)	50.0 1.7 (1.74–1.7)
No. reflections	37,520
* R*_work_/*R*_free_	0.138 (0.296)/ 0.203 (0.430)
No. atoms	
Protein	3311
Ligand/ion	148
Water	857
* B*-factors	
Protein	11.4
Ligand/ion	17.0
Water	25.5
R.m.s. deviations	
Bond lengths (Å)	0.018
Bond angles (°)	1.904

Structure was generated from a single crystal. Statistics for the highest resolution bin are in parenthesis

The backbones of the tunnel-enclosing loops (B2, B3, and A3 in particular) in *Pf*Cel7A overlay that of *Tr*Cel7A quite closely. However, the substrate-binding tunnel of *Pf*Cel7A tends to expose the substrate to solution more as a result of a tyrosine to alanine substitution (*Tr*Cel7A Tyr371 is replaced by *Pf*Cel7A Ala375). As a result, the exposure of a bound ligand to solvent is intermediate between *Tr*Cel7A and *Phanerochaete chrysosporium* Cel7D (*Pc*Cel7D, Supplementary Fig. 4). *Pf*Cel7A possesses the B3 loop (aka the “exo loop”) that *Pc*Cel7D lacks^15^. This structural feature has been demonstrated to have functional significance, impacting processivity, product inhibition, and activity on cellulose^52^. A significant difference in the main binding tunnel loops between *Tr*Cel7A and *Pf*Cel7A is that of a shortened A1 loop in *Pf*Cel7A, corresponding to a three-residue deletion in *Pf*Cel7A (A100, Q101, K102 *Tr*Cel7A numbering). In GH7 crystal structures, there are three basic structural modes for this loop, with *Tr*Cel7A possessing the intermediate length loop. The elongated A1 loop is exhibited by *Heterobasidion irregulare* Cel7A (*Hir*Cel7A)^53^, *Limnoria quadripunctata* Cel7B (*Lq*Cel7B)^14^, *Melanocarpus albomyces* Cel7B (*Ma*Cel7B)^16^, and *Humicola grisea* var. *thermoidea* Cel7A (*Hg*Cel7A)^54^*. Pf*Cel7A features the shortest of these modes, also displayed by *Pc*Cel7D, *Talaromyces emersonii* Cel7A (*Te*Cel7A)^13^, and *Trichoderma harzianum* Cel7A (*Th*Cel7A)^22^. In *Tr*Cel7A, this loop includes two glutamine residues, both of which could interact with the crystalline cellulose surface and one of which is within hydrogen bonding distance of the extracted cellulose chain. *Pf*Cel7A lacks these interactions (one is deleted and the other is repositioned due to the shorter loop), in addition to losing a salt bridge between Lys102 and Glu408, the latter of which is located on the A2 loop.

Relatively close to the tunnel entrance is another potentially significant difference: *Pf*Cel7A lacks a disulfide bridge that *Tr*Cel7A possesses. In all, *Tr*Cel7A has ten total disulfide bridges and *Pf*Cel7A contains nine. The tenth disulfide bond between Cys4 and Cys72 (*Tr*Cel7A) is relatively rare amongst GH7 members with solved structures, exhibited only in *Tr*Cel7A, *Th*Cel7A, and *Hg*Cel7A.

### Construction of a CD mutant library

Subsequent to solving the *Pf*Cel7A crystal structure, we identified seven areas of the enzyme wherein the structure differed significantly from that of *Tr*Cel7A. Each of these motifs was swapped into the corresponding region of the *Tr*Cel7A parent. A complete description of these catalytic domain mutants (T1–T7) is found in Supplementary Figs. 5 and 6.

**Fig. 4:**
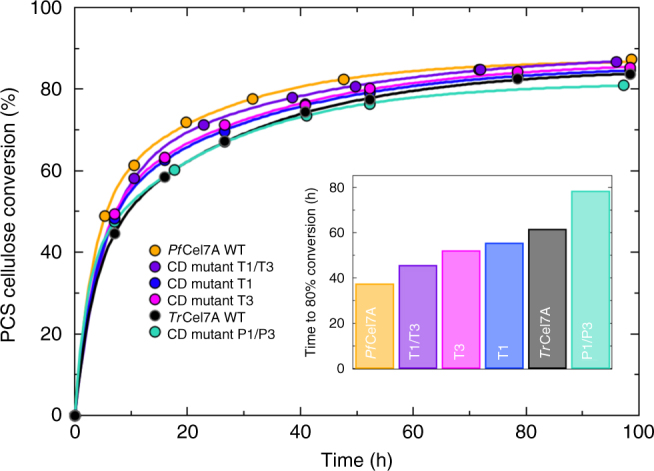
Selected catalytic domain (CD) mutant activity data. T1 and T3 (*Tr*Cel7A parent enzyme) show slight improvement in activity on pre-treated corn stover (PCS) over WT *Tr*Cel7A, whereas combining these into the double mutant T1/T3 demonstrates activity comparable to WT *Pf*Cel7A. Experiments were performed in triplicate; error bars represent the SEM and are smaller than the data markers

Two of these CD mutants, namely T1 (removal of the tenth disulfide bridge by C4G and C72A mutations) and T3 (three-residue deletion in the A1 loop), exhibited higher activity on PCS than the WT *Tr*Cel7A parent enzyme (Fig. 4). These two mutations (T1/T3) were then combined into the same mutant. Interestingly, with the mutation of two localized regions of the parent enzyme *Tr*Cel7A, hydrolytic activity nearly matches that of the *Pf*Cel7A WT, representing an improvement of >60% in terms of the time to 80% conversion (as measured by a double exponential fit to the conversion as a function of time, Supplementary Fig. 7).

We also performed the converse experiment; that is we introduced the features of *Tr*Cel7A corresponding to CD mutants T1 and T3 into the *Pf*Cel7A parent enzyme to see whether the performance of *Pf*Cel7A could be significantly diminished with the introduction of these limited mutations. In particular, we created CD double mutant P1/P3. As before, we see that with these two limited modifications, the activity can be modulated back to that of the WT *Tr*Cel7A (Fig. 4). Whether introducing these two features (into *Pf*Cel7A) or removing them (from *Tr*Cel7A), it is clear that activity can be modulated between the upper bound of WT *Pf*Cel7A and the baseline of WT *Tr*Cel7A.

### Mechanistic insight via molecular simulations

To further understand the significant performance differences from relatively modest structural changes, we performed 1 µs molecular dynamics (MD) simulations of WT *Pf*Cel7A CD, WT *Tr*Cel7A CD, and the CD double mutant T1/T3 (*Tr*Cel7A parent) CD in solution and the latter two enzymes with the *Tr*Cel7A linker/CBM complexed on a cellulose microfibril.

**Fig. 5:**
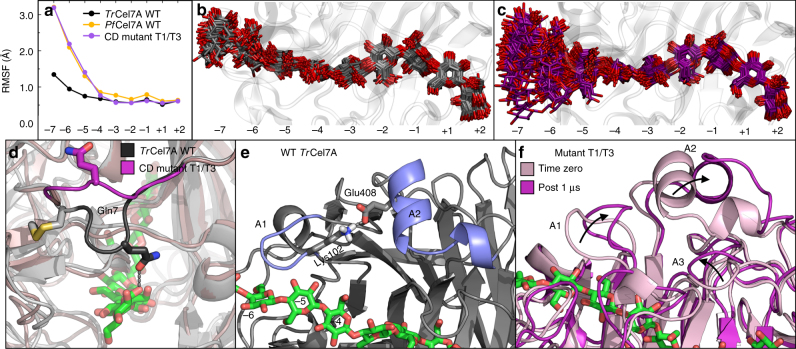
Mechanistic insight from molecular simulations. **a** The RMSF profile for the glucosyl residue at each binding site in solution. This contrast in flexibility can be seen **b** in the WT *Tr*Cel7A, and **c** in the mutant enzyme, particularly at the −5, −6, and −7 subsites. The ligand is shown every 8 ns over 1 µs. **d** Whereas Gln7 in WT *Tr*Cel7A maintains a hydrogen bond with the glucosyl moiety in the −7 binding site, the mutant is more flexible, essentially abolishing this enzyme-substrate interaction at the tunnel entrance. **e** In the WT enzyme, a persistent salt bridge forms between Lys102 (A1 loop) and Glu408 (A2 loop). **f** The deletion of Lys102 results in the loss of this salt bridge in the mutant enzyme, resulting in significant rearrangements for loops A1, A2, and A3

The effect of the disulfide removal (C4G, C72A) is observed most clearly in the solution simulations. The tunnel entrance (roughly residues 3–12) is clearly more flexible in the double mutant, as illustrated by comparing the RMSF profiles (Supplementary Fig. 8). This increased flexibility is unique to the tunnel entrance—no other region of the enzyme that interacts with the substrate exhibits any significant difference. As a result, the ligand in the mutant enzyme is considerably more flexible and explores a much wider range of space near the tunnel entrance than WT *Tr*Cel7A (Fig. 5a–c). The increased flexibility of the enzyme and ligand upon disulfide removal can, at least in part, be attributed to the loss of a stable hydrogen bond in WT *Tr*Cel7A between Gln7 and the glucose in the −7 binding site (Fig. 5d). In the absence of the disulfide, the short loop containing Gln7 moves away from the substrate, the hydrogen bond is no longer present, and the ligand moves more freely. As expected with increased flexibility, ligand solvation also increases at the binding tunnel entrance. The three binding sites nearest to the tunnel entrance have, on average, 2–3 more water molecules nearby in the CD mutant T1/T3 (near the levels of *Pf*Cel7A WT) than their counterparts in *Tr*Cel7A WT (Supplementary Fig. 9).

The effect of the A1 loop shortening (deletion of Ala100, Gln101, Lys102) is seen in the surface simulations, where a “cascade effect”, leads to large-scale rearrangements of loops A1, A2, and A3 (Fig. 5f), making other substrate-enclosing loops (e.g., B1, B2, A2, A4) significantly more flexible (Supplementary Fig. 10). The trigger for these widespread changes is likely the loss of the stabilizing salt bridge present in the WT between Lys102 (A1 loop) and Glu408 (A2 loop) (Fig. 5e), which leads to large-scale movements of these loops to new equilibrium positions leading to the “opening” of the A3 loop as well (Fig. 5f). In addition to the lost salt bridge, both the solution and surface simulations reveal a stable hydrogen bond between the sidechain oxygen of Asn103 and the O_2_ hydroxyl group of the glucose in the −5 binding site in WT *Tr*Cel7A that is considerably more transient in the double mutant.

## Discussion

GH7 CBHs are the cornerstone of modern industrial enzyme cocktails to deconstruct cellulose. Yet, despite a wealth of recent structural data, only a few self-consistent studies to date have directly related enzyme performance on relevant substrates at industrially relevant conversions to structural features. Recent years have seen many studies published on the details of the catalytic cycle of processive cellulases (often utilizing *Tr*Cel7A), shedding light on the rate-limiting step (RLS) in deconstructing cellulose under various conditions^8,17,21,32,48,49,55,56^. Most cellulase engineering efforts to date have, however, not targeted a specific step of the processive cycle (namely, the RLS), but have instead tended to target more thermostable enzymes. Several such studies have addressed reducing end specific CBHs using an array of tools including biased DNA shuffling^41^, rational mutagenesis^18^, consensus analysis of multiple sequence alignments^57^, SCHEMA^38,58^, and directed evolution^20,59^; these often achieved higher activity due to higher operating temperature, and even enhanced synergism in an enzyme cocktail^39^.

In the current study, we employed a chimera library approach both at the domain (CBM, linker, CD) level and a more localized level, demonstrating that no single domain is fully responsible for the activity enhancement of WT *Pf*Cel7A over WT *Tr*Cel7A. This superiority was indicated previously, albeit at short incubation times and low conversions (<25%)^60^, but is here demonstrated on real biomass to industrially relevant conversions in a synergistic enzyme cocktail. Elucidation of the crystal structure of the *Pf*Cel7A CD indicates local regions of structural variation from *Tr*Cel7A, representing possible sources for the superior activity of the *Pf*Cel7A CD. By targeting these regions to produce a CD mutant library, it was found that two local structural changes—removal of a disulfide bond and shortening of a tunnel-enclosing loop, both near the tunnel entrance—impart a significant improvement in performance on biomass when employed in an enzyme cocktail. MD simulations shed light on the structure-function link by revealing increased ligand flexibility at the binding tunnel entrance as well as modulation of loop position and increased flexibility—characteristics likely to increase the ability to dissociate from a cellulose chain.

Kont et al.^51^ recently reported “inter-domain synergism” in *Tr*Cel7A wherein the effect of a W38A mutation at the CD tunnel entrance on the association constant (*k*_on_) was strongly dependent on whether the CBM-linker were attached, indicating some “compensation” between the variants in different domains. Our domain-swapping experiments indicate that chimeras with the *Pf*Cel7A CD tend to be the most active, but not uniformly so, demonstrating interrelatedness between domains, the source of which is not obvious. As such, the results presented in Fig. 2b suggest not only that significant performance improvements can be made to cellulases based on natural diversity screening and chimera library generation, but also motivate both further domain-swapping experiments in Family 7 (and other GH families) and further biophysical studies into the mechanism of multi-modular processive cellulases.

The rate-limiting step of cellulose deconstruction by processive GHs in the absence of synergistic polysaccharide-active enzymes such as non-reducing end specific CBHs, endoglucanases, and lytic polysaccharide monooxygenases (LPMO) is the dissociation rate (*k*_off_)^48,61–64^. Experimentally, we have mapped the superior activity of the *Pf*Cel7A CD (vs. *Tr*Cel7A) to two very localized regions of the structure and demonstrated via MD simulations enhanced flexibility of both substrate and enzyme in the CD mutant T1/T3, flexibility which could have a marked effect on *k*_off_. Previous work has shown that rigidifying a substrate-enclosing loop (B3 in this case) slightly decreases CBH processivity^52^, suggesting a proportional increase in *k*_off_^65^, however, other modifications were also observed, including the loss of multiple hydrogen bonding interactions between the exo-loop and the substrate at the active site. We propose here that the most natural connection is that more flexible substrate-enclosing loops will likely, in general, result in a more efficient enzyme given its increased ability to dissociate when encountering an obstacle^48,49^. These connections are supported by recent mutagenesis experiments targeting loop regions in *Te*Cel7A that showed enhanced cellulose hydrolysis when loop–loop and loop–substrate interactions were decreased^66^.

Connecting cellulase structural features with hydrolytic function is essential to the future success of engineering efforts, and ultimately to the development of industrial biochemical processes to produce fuels, chemicals, and materials from biomass. Going forward, more direct, self-consistent comparisons of the type presented here will be critical to successfully establishing this enhanced structure-function understanding. In addition, these comparisons must take place in a context that is as industrially applicable as possible, including relevant biomass types and loadings, in a synergistic cellulase cocktail, with assays conducted to high conversion extents. The current work has attempted to fulfill this, but even so, some aspects may warrant subsequent study such as whether a more complex cellulase background (e.g., in the presence of non-reducing end specific cellulases, additional endoglucanases, and LPMOs) could affect the results. Questions of this nature can only be resolved by further comparative structure-function studies at industrially relevant conditions.

## Methods

### Plasmids

All cellobiohydrolase (CBH) enzymes were expressed using the pTrEno plasmid^45^. The pTrEno vector is essentially an Enolase promoter driven expression integration vector selected for with hygromycin resistance. CBH genes were synthesized and cloned into the *PacI and XbaI* sites of pTrEno using either traditional ligation based cloning or using Gibson Assembly (New England Biolabs). To prep these plasmids for transformation the pTrEno-based plasmids containing the CBH gene were cut with *SbfI* and *XhoI* and integrated randomly into the genome.

### Transformation of *T. reesei*

We used transformation by electroporation. Spores of AST1116 (QM6A CBH1Δ::AMDS) from a frozen glycerol stock were plated onto Potato Dextrose (PD) plates and allowed to grow for 3–5 days in a lighted incubator until the plates were green from abundant conidial spores. Spores were collected by spreading sterile deionized water (diH_2_O) onto the plates and recovering the spores in centrifuge tubes. Cells were adjusted to an OD_600_ of 0.03 in water and then 100 mL of cells were spread onto PD plates. These plates were grown in a lighted incubator at 30 °C for two days. Spores were again harvested by spreading 5 mL of ice-cold sterile diH_2_O and using a sterile spreader to liberate the conidial spores from the mycelia. Spores were collected in 50 mL conical centrifuge tubes and were centrifuged for 5 min at 2000 × *g*. Spores were washed twice with ice-cold sterile diH_2_O and twice with cold sterile 1 M sorbitol repeating the above centrifuge conditions in between each wash. Spores were suspended in cold 1 M sorbitol to a final OD_600_ of 2. Spore aliquots were frozen on dry ice and stored indefinitely at −80 °C.

Frozen spores aliquots were thawed on ice. To 75 μL spore suspension we added ~1 μg of linearized DNA (see Plasmids section above) in a microcentrifuge tube. The DNA spore mixture was mixed and then transferred to a 0.1 cm gap electrocuvette (BioRad). These were then electroporated using the following settings on a BioRad Gene Pulser: 1.8 kV, 800 Ω, 25 μF. Immediately after delivering the pulse the cuvettes were placed back on ice for 5–10 min. Following this incubation, 1 mL of Complete Media with Lactose [“CML” per 1 liter: 5 g yeast extract, 5 g tryptone, 10 g lactose, autoclave, then cool, add 50 mL Clutterbucks Salts (per liter: 120.0 g NaNO_3_, 10.4 g KCl, 10.4 g MgSO_4_, 30.4 g KH_2_PO_4_)] is added to the cuvette and then transferred to sterile tissue culture plates with either 6, 12, or 24 wells and allowed to recover overnight (~ 18 h) at room temperature. Lactose is used for convenience because cells can recover overnight and the mycelial growth is minimal by the morning. In the morning, 200 μL of cells are plated onto PDHX plates or PD plates supplemented with 100 μg/mL hygromycin and 0.1% v:v IGEPAL CA-630 (Similar to Triton X, Sigma-Aldrich). Plates were incubated at 30 °C for 2–3 days until colonies appeared.

### Expression screening

Colonies were picked by coring out the agar using the wide end of a sterile 20–200 μL pipette tip and inoculating 2–5 mL of a modified version of Mandels and Andreotti medium^67^ with glucose (“MAG”, per liter 20 mL 50× Mandel’s salts to 500 mL, 5 g tryptone, H_2_O to 720 mL, pH to 5.5 with KOH, autoclave, add 10 mL micronutrient solution, add 2.7 mL 1 M CaCl_2_, add 250 mL 20% glucose). 50× Mandel’s salts per liter: 100 g KH_2_PO, 70 g (NH_4_)_2_SO_4_, 15 g urea, 15 g MgSO_4_.7H_2_O, pH to 5.5 with KOH. Micronutrient solution per liter: 500 mg FeSO_4_.7H_2_O, 160 mg, MnSO_4_.H_2_O, 140 mg ZnSO_4_, 200 mg CoCl_2._ Picked colonies were incubated in sterile tissue culture plates statically at 30 °C in a lighted incubator which allows for mycelial mats to form on the surface of the media; cellulases are secreted into the medium. After 2–3 days of growth, broth supernatants were screened by immunoblotting using an anti-*P. funiculosum* Cel7 antibody (2.4 mg/mL—custom made in rabbits by Robert Sargeant, 655 Ash Street, Ramona CA 92,065, and used at 1:2000 dilution), and an alkaline phosphatase conjugated goat anti-rabbit IgG antibody (0.6 mg/mL—purchased at Thermo Scientific product #31342 and used at 1:2000 dilution). For positive reacting clones, the mycelial mats were lifted from the suspension and placed onto PDH plates and allowed to sporulate for 2–3 days as described above. These spores were then restreaked onto PDHX plates for single colonies. Several single colonies were then grown up again in MAG and confirmed for expression.

### Growth conditions for *T. reesei* expression strains

Recombinant strains were streaked onto PD agar plates and allowed to grow 2–3 days until dense sporulation was achieved. A ~0.5 cm plug was extracted from the plate and deposited into 1 L of liquid growth media in a 2.8 L shake flask. The growth media consisted of Mandel’s growth media^67^ with 5% glucose as the carbon source, and 0.5% tryptone added. The culture was grown at 28 °C with agitation for 24 h, after which the entire 1 L was transferred to 7 L of the same media, in a bioreactor. The bioreactors are 15 L working volume vessels manufactured by New Brunswick and controlled via New Brunswick’s BioFlo310 system. The total of 8 L was grown with mixing at 300 RPM via dual down-flow marine style impellers, purged with 1.5 vvm of filtered air, kept at a strict 28 °C, and pH controlled at 4.8 The acid and base used for pH control were HCl and KOH, respectively. The cell culture was grown for 48 h, after which the entire culture broth was drained, filtered through nylon to remove all cell mass, and concentrated via tangential flow filtration with a 10 kDa MWCO (GE Health Sciences) concentrator. The concentrated broth was buffer-exchanged into 20 mM Bis-Tris buffer, pH 6.5, and brought up to ~200 mL.

### Purification of proteins

Culture supernatants were collected as follows. Mycelial mass was removed by gravity filtration using Miracloth (EMD Biosciences, Gibbstown, NJ). The supernatant obtained was passed under vacuum through a series of glass fiber filters with descending pore sizes ranging from 2.7 to 0.7 µm (Millipore, Billerica, MA). This was followed by vacuum filtration through 0.45 µm bottle-top filtration devices (Nalgene, Rochester, NY). After filtration, supernatants were concentrated to ~2% of the original volume and exchanged into 20 mM Bis-Tris buffer, pH 6.5 using a 10 kDa MWCO hollow fiber tangential flow filtration system (GE Life Sciences QuixStand).

The concentrated supernatants were amended to 2 M with solid (NH_4_)_2_SO_4,_ loaded onto a Tricorn 10/100 column (GE Healthcare, Piscataway, NJ) packed with Source 15Phe Hydrophobic Interaction Chromatography medium (GE Healthcare), and eluted using a 25 column volume linear gradient of 20 mM Bis-Tris, pH 6.5, 1.8 M (NH_4_)_2_SO_4,_. Fractions were assayed for Cel7A activity by adding 25 µL of each fraction to 100 µL of 2.0 mM *p*-nitrophenol-β-lactopyranoside (*p*NPL; Sigma-Aldrich, St Louis, MO) in 50 mM Sodium Acetate, pH 5.0 in a microtiter plate. Reactions were incubated for 30 min at 45 °C and quenched by addition of 100 µL 1.0 M sodium carbonate. Absorbance was read at 405 nm and fractions with significant *p*NPL activity were pooled and examined by SDS–PAGE and western blotting using polyclonal antibodies raised against the catalytic domain of *T. reesei* or *P. funiculosum* Cel7A, as required. Pooled peaks containing Cel7A were concentrated by centrifugal ultrafiltration using a 10 kDa PES membrane (Viva Science) and desalted into 20 mM Bis-Tris pH 6.5 using two Hi-Prep 26/10 desalting columns in series. The resultant protein solution was loaded onto a Tricorn 10/100 column packed with Source 15Q anion-exchange medium (GE Healthcare) and eluted with a 50 column volume linear gradient of 0–1.0 M NaCl in 20 mM Bis-Tris pH 6.5. Fractions were assayed and visualized as above. Active fractions were pooled and amended to 2.0 M (NH_4_)_2_SO_4,_ and loaded onto a Tricorn 10/100 Source 15Iso hydrophobic interaction chromatography column, and eluted with a 1.8–0.0 M (NH_4_)_2_SO_4,_ gradient in 20 mM Bis-Tris pH 6.5. Active fractions were pooled and concentrated to a volume <13 mL in 10 kDa MWCO Vivaspin-20 centrifugal concentration device and loaded onto a HiLoad Superdex75 26/60 size exclusion column and eluted at 2.0 mL/min in 20 mM Sodium Acetate, pH 5.0, 100 mM NaCl. Fractions were assayed and visualized as above. Active fractions were pooled, concentrated as above, and protein concentration was determined as a function of absorbance at 280 nm using extinction coefficients specific to each protein sequence as calculated using Swiss-Prot.

### Activity assays

CBH activity was measured as the saccharification of the cellulose fraction of a sample of a standard dilute-acid-pretreated corn stover by a given CBH species (loaded at 28 mg CBH per g of biomass glucan) when used as part of a cocktail also containing two other glycolytic enzymes at standard loadings: (1) the endoglucanase *Acidothermus cellulolyticus* E1 (Cel5A, mutant Y245G, catalytic domain only)^68^ at 1.894 mg/g of biomass cellulose to provide synergistic cellulolytic action with the CBH and (2) the chromatographically purified β-glucosidase from *Aspergillus niger*, loaded at 0.5 mg/g biomass cellulose to minimize product inhibition by cleaving product cellobiose to glucose.

The standard biomass substrate used in the activity assays was NREL dilute-acid-pretreated corn stover^69^, (“pre-treated corn stover”, PCS) P050921, produced in a vertical pulp digester supplied by Sunds Defibrator (now Metso Corporation, Helsinki) with a residence time of ~1 min at 190 °C with 0.45 g H_2_SO_4_ per g dry biomass at 30% solids loading, to yield a material with 59.1% glucan, 5.1% xylan, and 25.3% lignin. The PCS was washed first with water and then with 20 mM acetic acid/sodium acetate buffer, pH 5.0, until the pH of the (buffer) decantate was within 0.03 units of 5.00. From a slurry of this washed biomass material (~9 mg biomass per mL of pH 5.0, 20 mM acetate buffer containing 0.02% sodium azide to retard microbial growth), a series of biomass substrate aliquots were prepared in 2.0 mL HPLC vials, in such a way that each vial contained 8.5 mg biomass cellulose (which, given that the glucan content of this batch of PCS is 59.1%, required 14.38 mg of biomass per digestion vial). Biomass dry weights for each batch of assay vials were verified by dry-weight determinations on a group of five samples co-pipetted into pre-tared vials. The acceptable relative standard deviation for a batch of biomass assay aliquots was 1% or less, with a preferred value of 0.8% or less. Adjustment of these biomass assay aliquots to a 1.7 mL final volume resulted in a cellulose concentration of 5 mg/mL.

CBH assays were conducted in triplicate vials at 40 °C, pH 5.0 in 20 mM azide-containing acetate buffer, with continuous mixing by inversion at 10 RPM while immersed in a water bath. At various times during the digestion, the digestion vials were removed from the rotator and representative 100 μL samples containing both solids and liquid were removed from the well-stirred contents and diluted 18-fold into glass HPLC vials. The primary digestion vials were immediately resealed and returned to the rotator in the assay (40 °C) water bath to allow the assay digestions to continue. The vials containing the withdrawn and diluted samples of digestion mixture were then crimp-sealed and immersed in a boiling-water bath for 10 min to denature the enzymes and terminate the reaction. The contents of the boiled time-sample vials were then syringe-filtered (0.2-micron Acrodisc) into a third set of vials for sugar analysis by HPLC on a BioRad HPX-87H column eluted at 55 °C with 0.01 N H_2_SO_4_ at 0.6 mL/min with refractive-index detection. Values for individual sugar concentrations in the digestion vials were back-calculated from the values measured by HPLC, and then used to construct saccharification progress-curves in terms of percent of conversion of biomass cellulose.

### *Pf*Cel7A and *Tr*Cel7A enzyme activity on *p*NPL

*V*_max_ and *K*_m_ were determined using *p*-nitrophenyl-β-D-lactopyranoside (*p*NPL, Sigma) as substrate. 0, 0.13, 0.26, 0.66, 1.3, 2.6, 5.3, 6.6 mM *p*NPL (150 μL) was incubated with each CBH (25 μL) at 40 °C in 20 mM acetate buffer pH 5.0, 100 mM NaCl, in 96-well microtiter plates. Reactions were quenched by the addition of 25 μL of 1.0 M sodium carbonate after 30 min. The absorbance values were converted to a molar concentration using *p*NPL standard curve (standards concentration range: 0–250 μM). Activity values were fit to the Michaelis–Menten expression using GraphPad Software (La Jolla, California USA). All reactions were carried out in triplicate.

### *Pf*Cel7A and *Tr*Cel7A pH profile measurements

pH profiles were generated in duplicate by incubating 1.6 mM *p*NPL with 0.66 μM indicated enzyme in 150 μL of MacIlvaine buffer (citrate-phosphate-NaCl) with a constant conductivity between 19 and 21 mS/cm. pH measurements were completed in a 96-well plate at pH 2.9, 3.9, 5.0, 6.1, 7.1 and 8.3 at 40 °C for 0, 1, 5, 10, 20, and 30 min. The reaction was stopped by the addition of 25 μL of 1.0 M sodium carbonate and the A_405_ was determined by a colorimetric microtiter plate reader. The absorbance values were correlated to *p*NPL concentration via *p*NP standards and the pH profile graphs were generated by plotting the linear rate of *p*NPL turnover as a function of pH.

### *Pf*Cel7A and *Tr*Cel7A temperature profiles

Temperature profiles were generated in duplicate by incubating 1.6 mM *p*NPL with 0.66 μM enzyme in 2.0 mL of 20 mM acetate buffer pH 5 or pH 4, 100 mM NaCl and 0.02% NaN_3_. Reactions were incubated at 30, 35, 40, 45, 50, 55, 60, and 65 °C in a water bath. At 0, 1, 5, 10, 20, and 30 min, 150 μL aliquots were removed from reaction vial to a 96 well plate containing 25 μL of 1.0 M sodium carbonate. Absorbance values were measured at 405 nm and temperature profile graphs were generated by plotting the linear rate of *p*NPL turnover as a function of temperature.

### Differential scanning calorimetry

Thermal stability was evaluated by differential scanning microcalorimetry (DSC) using a MicroCal model VP-DSC calorimeter (MicroCal, Inc., Northhampton, MA). Data were collected by Origin for DSC software (MicroCal). Samples contained 50 μg/mL protein at pH 5.0 in 20 mM acetate, 100 mM NaCl. Calorimeter scan rate was 60 °C/h with a range of 30–110 °C.

### Deglycosylation

Before crystallization the purified *Pf*Cel7A was deglycosylated with jack bean alpha-mannosidase. For 1 mg of *Pf*Cel7A 40 units of alpha-mannosidase (Sigma-Aldrich, St. Louis, MO, USA) were used in a buffer containing 100 mM acetate pH 4.5, 100 mM NaCl, 5% glycerol, and 1 mM zinc acetate. The sample was then incubated at 37 °C in a rotating incubator for 24 h. After deglycosylation the alpha-mannosidase and partially deglycosylated *Pf*Cel7A were removed by repeating the anion exchange chromatography as described above and size exclusion chromatography using a 26/60 Superdex 75 column in 20 mM acetate pH 5.0, 100 mM NaCl, and 50 mM cellobiose.

### Crystallization

Crystals of the deglycosylated *Pf*Cel7A were initially detected with sitting-drop vapor diffusion crystallization using a 96-well plate, Grid Screen Salt HT from Hampton Research (Aliso Viejo, California, USA) and setup by a Phoenix crystallization robot (Art Robbins Instruments, Sunnyvale, California, USA). For the final data collection crystals were grown using a sitting drop setup with 50 µL of well solution in the reservoirs and 0.5 µL of well solution and 1.5 µL of protein solution in the drop. The crystals grew at 20 °C in 0.27 M sodium phosphate monobasic monohydrate and 1.53 M potassium phosphate dibasic pH 4.0 as the well solution. The protein solution contained 15.5 mg/mL of protein in 20 mM acetate pH 5.0, 100 mM NaCl, 50 mM cellobiose, and 5 mM cellohexaose.

### Data collection and processing

The *Pf*Cel7A crystal was flash frozen in a nitrogen gas stream at 100 K before data collection at 1.54178 nm wavelength using an in-house Bruker X8 MicroStar X-Ray generator with Helios mirrors and a Bruker Platinum 135 CCD detector. No cryoprotectants were used. Data were indexed and processed with the Bruker Suite of programs version v.2013.3 (Bruker AXS, Madison, WI).

### Structure solution, refinement, and structure analysis

Intensities were converted into structure factors and 5% of the reflections were flagged for *R*_free_ calculations using programs F2MTZ, CTRUNCATE, CAD, and Unique from the CCP4 package of programs^70^. The program MOLREP^71^ version 11.2.05 was used for molecular replacement with *Talaromyces emersonii* Cel7A (PDB entry 3PL3) as the search model. Refinement and manual correction were performed using REFMAC5^72^ version 5.8.0073 and Coot^73^ version 0.7.1. MolProbity^74^ version 4.1 was used to analyze the Ramachandran plot and root mean square deviations (RMSD) of bond lengths and angles were calculated using ideal values of Engh and Huber stereochemical parameters^75^. According to Ramachandran plot statistics all residues were in the allowed regions, 98.1% were in the favored region and there were no outliers. Average B-factors, were calculated using program ICM version 3.8–0 (Molsoft LLC, La Jolla, CA). Programs Coot and PyMOL (http://www.pymol.org) were used for comparing and analyzing structures. The data collection and refinement statistics are shown in Table 1.

As discussed in the main text, the structure of the *Pf*Cel7A CD was solved to 1.70 Å resolution. As with essentially all GH7 family members with published crystal structures, the backbone of *Pf*Cel7A overlays that of *Tr*Cel7A quite closely. In the main text, we discussed various structural features wherein *Pf*Cel7A varies from *Tr*Cel7A; we continue that discussion here. Another loop difference occurs on the “top” side of the enzyme (opposite the crystalline cellulose surface). This loop has three modes within GH7 crystal structures. *Tr*Cel7A possesses the shortest of these modes, whereas *Pf*Cel7A has the longest, a feature it shares with *Pc*Cel7D, *Hir*Cel7A, *Re*Cel7A, and *Tr*Cel7B.

At the binding tunnel entrance Tyr6 of *Pf*Cel7A sits above the decrystallized cellulose chain just beyond the –7 binding site (where Trp40 stacks on that glucosyl moiety). There is a high degree of variability at this residue in GH7 members with solved structures, and *Pf*Cel7A is the only of these to exhibit Tyr here. The space occupied by the side chain of *Pf*Cel7A Tyr6 is similar to that of *Pc*Cel7D Tyr47 and *Tr*Cel7B Tyr46.

The four tryptophan residues that line the binding tunnel in GH7 CBHs are also found in *Pf*Cel7A (residues 40, 38, 371, and 380, which stack on the −7, −4, −2, and +1 glucosyl rings, respectively), and their positions overlay those of *Tr*Cel7A (as well as every other GH7 CBH) nearly perfectly. In addition, the catalytic residues (nucleophile Glu209, acid/base Glu214, and “helper” Asp211) overlay those of *Tr*Cel7A quite closely. At the product sites, *Pf*Cel7A shares a unique feature with *Tr*Cel7A; whereas every other GH7 CBH has aspartate at residue 340 (*Tr*Cel7A numbering), *Tr*Cel7A, *Pf*Cel7A, and *Th*Cel7A have glycine. This acidic residue is within hydrogen bonding distance to the cellobiose product in the primed state close to the binding tunnel exit. Finally, the cellobiose captured in the product sites of the *Pf*Cel7A crystal structure is in “primed GEI” mode. In fact, the cellobiose product captured here is the closest to the tunnel exit that has been captured to date in a GH7 crystal structure.

### Molecular simulations

Molecular dynamics (MD) simulations were performed both in solution and on the surface of a cellulose microfibril.

Solution simulations were performed with WT *Tr*Cel7A, WT *Pf*Cel7A, and CD mutant T1/T3. In each case, a cellononaose ligand was bound in the active site of the CD. Each enzyme was solvated in a box of explicit water molecules of approximate dimensions 80 × 80 × 80 Å^3^. Sodium ions were added to neutralize the system. The total system size was ~52,000 atoms.

MD simulations on the surface a cellulose microfibril were performed for *Tr*Cel7A and CD mutant T1/T3. The construction of the surface simulations followed the protocol of Payne et al.^33^ The *Tr*Cel7A CD structure was taken from the Protein Data Bank 8CEL with a cellononaose ligand in the tunnel to form the catalytically active complex^12^. The cellulose microfibril is 28 units long with four layers of cellulose chains. The top layer comprises three chains. *Tr*Cel7A is complexed with an edge chain of the top layer on the hydrophobic face of cellulose. Each system contained ~116,000 atoms with dimensions of ~160 × 80 × 80 Å^3^.

The initial simulation of WT *Tr*Cel7A (fully glycosylated) began with the linker unbound and ~12 glucose residues of the complexed chain displaced from their crystal structure positions within the microfibril surface. The CD mutant T1/T3 was built from this system after 200 ns of unrestrained molecular dynamics. In this time period, several residues of the cellulose chain anneal back into the surface, leaving only nine glucose residues outside of the microfibril. The linker domain also binds to the cellulose surface within 200 ns. Thus, the mutant simulation begins with the linker bound and the complexed cellulose chain in a relaxed configuration. From this point, both systems were simulated for an additional 1 μs.

Additional details on the molecular simulations can be found in the Supplementary Methods.

### Data availability

*Pf*Cel7A crystal structure is available in the Protein Data Bank (PDB) under accession number 4XEB. The data that support the findings of this study are available from the corresponding author on reasonable request.

## Electronic supplementary material

Supplementary Information(PDF 11564 kb)
