# Supplementary material for: Engineering enhanced cellobiohydrolase activity
Source: Nat Commun. 2018 Mar 22;9:1186. doi: 10.1038/s41467-018-03501-8 (PMC5864845; doi:10.1038/s41467-018-03501-8)
Supplement: Supplementary file 1 — Supplementary Information(PDF 11564 kb) [file 41467_2018_3501_MOESM1_ESM.pdf]

Supplementary Information for

Engineering enhanced cellobiohydrolase activity

Larry E. Taylor II *et al*.

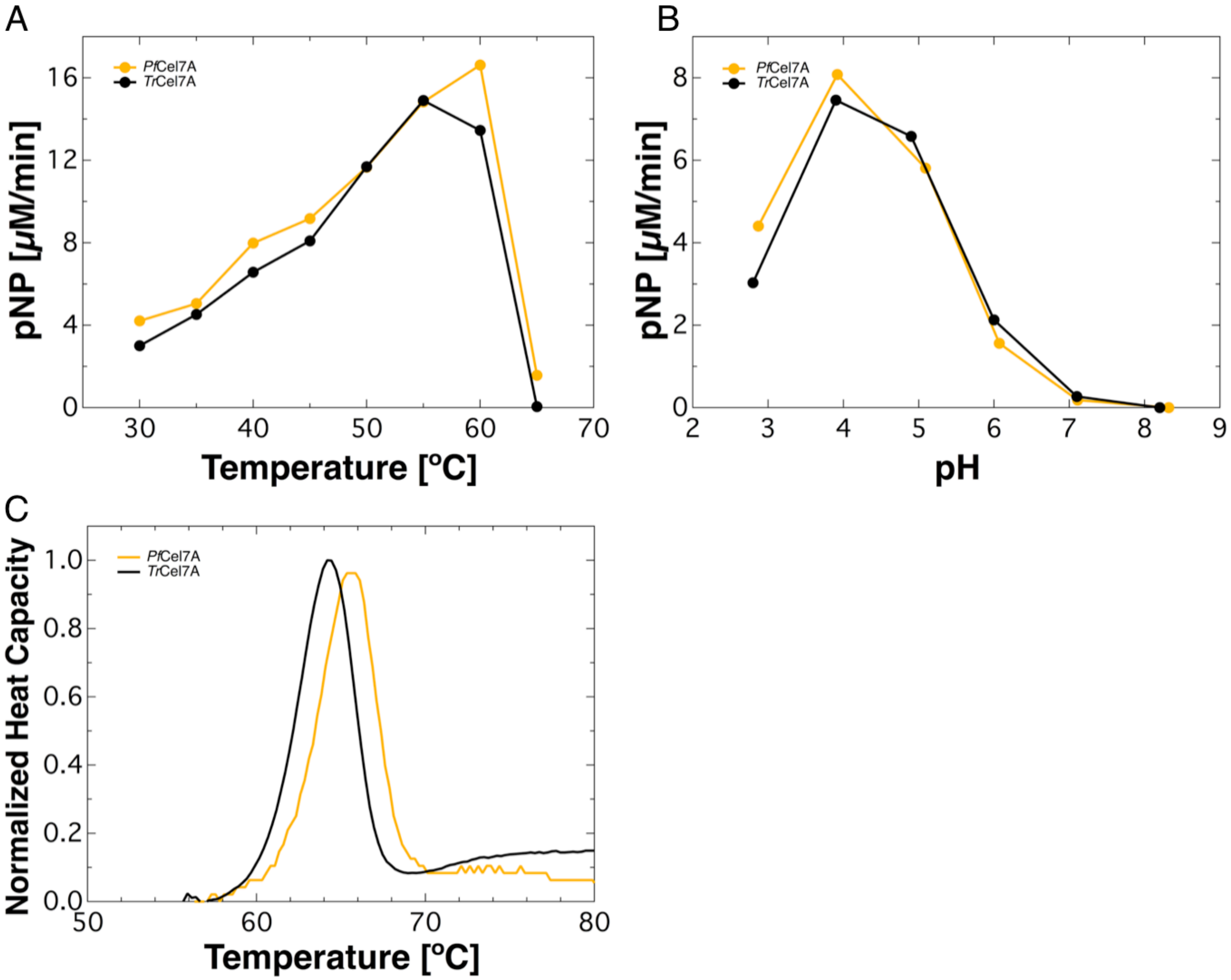

Supplementary Figure 1. Characterization of *Pf*Cel7A performance and comparison to *Tr*Cel7A. A) Activity comparison on 1.6 mM *p*NPL, pH 5.0. B) Activity comparison on 1.6 mM *p*NPL, 40°C. C) DSC traces computing denaturation temperature, T_{max}.

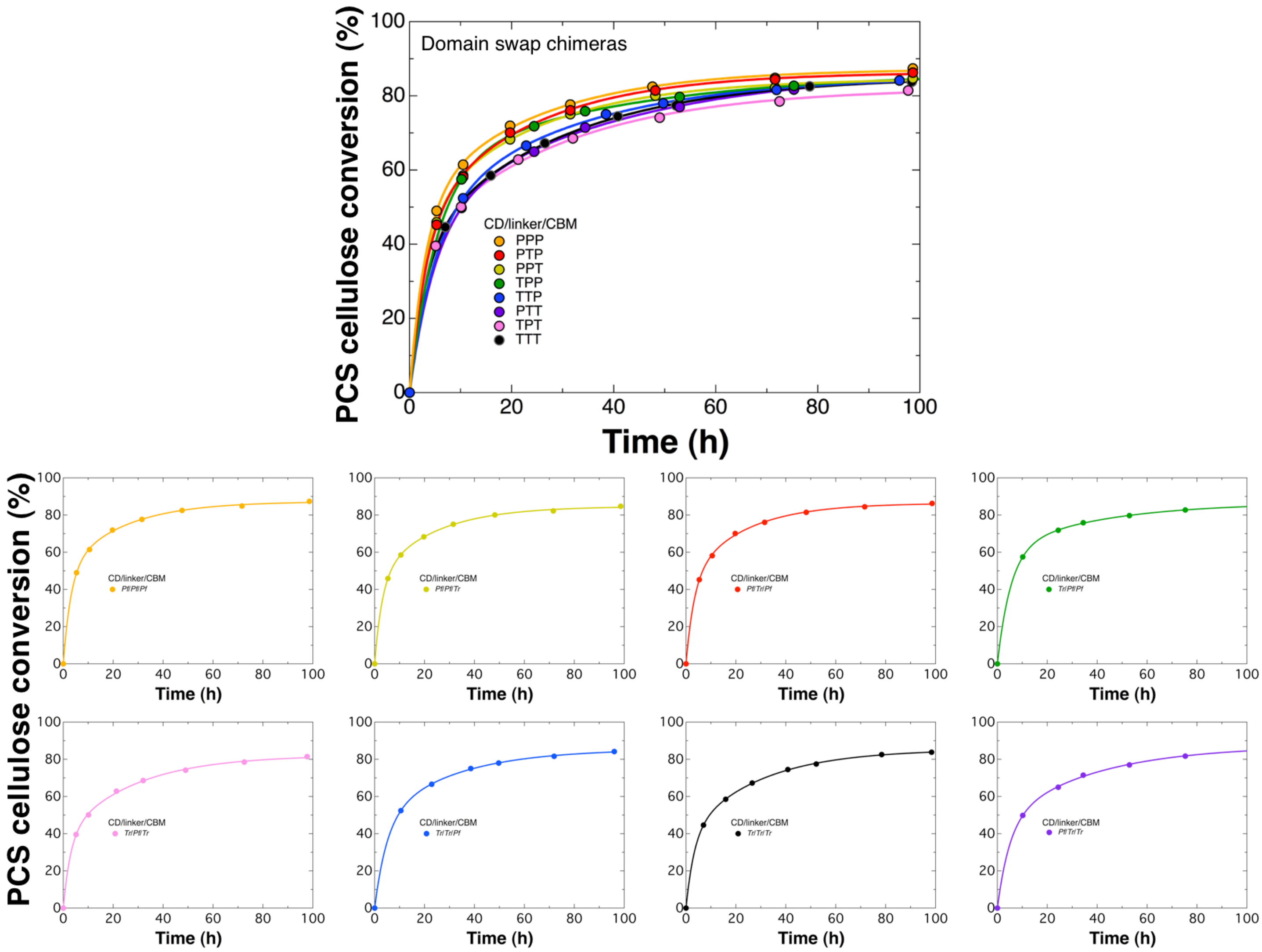

Supplementary Figure 2. Glucan conversion as a function of time on pre-treated corn stover (PCS) for the domain swap chimera library. Data points are shown as circles, and lines represent double-exponential fits to the data. The assays were performed at 40°C and pH 5.0. The time constants for these double exponential fits are represented in the main text Fig. 2B. Experiments were performed in triplicate; error bars represent the SEM and are smaller than the data markers.

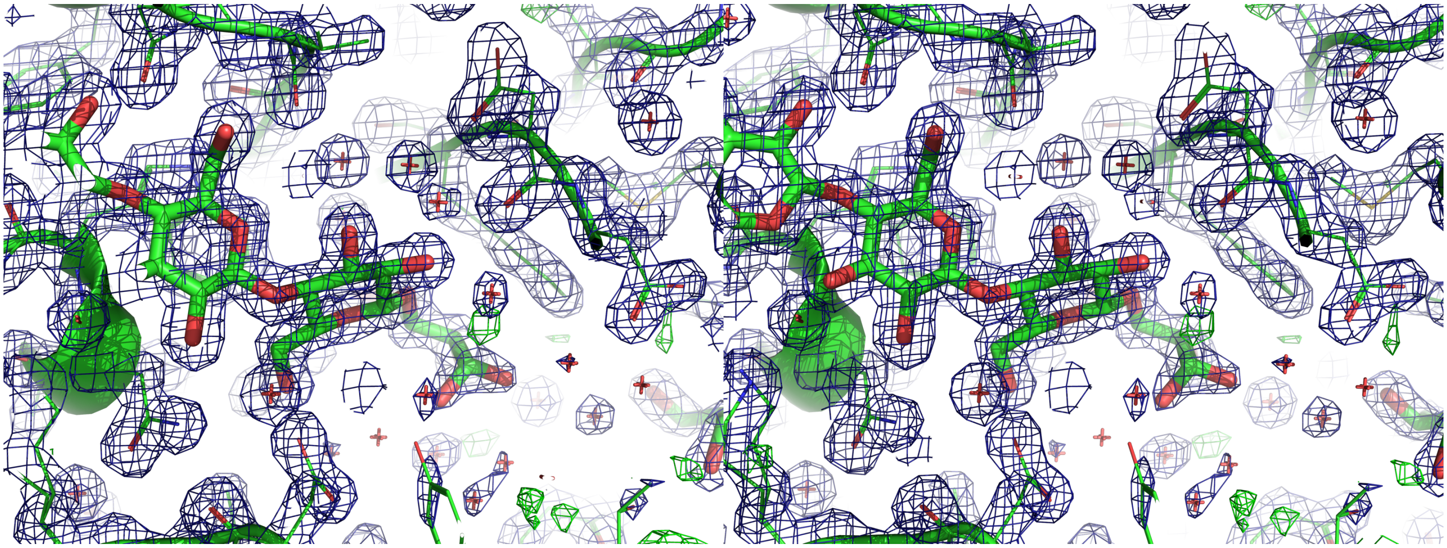

Supplementary Figure 3. Stereo image of a portion of the electron density map for the crystal structure of *Pf*Cel7A. Side-by-side stereo figure of the electron density near the active site showing part of the cellohexaose as sticks (green carbons, red oxygens) and the protein part as green ribbon/sticks (green carbons, red oxygens, blue nitrogens and yellow sulfurs). The blue 2fo-fc map is rendered at 1.3σ and the green fo-fc map at 2.9σ. Both maps were generated by REFMAC5[1] from the last refinement run of the structure and the figure was made using PyMol.[2]

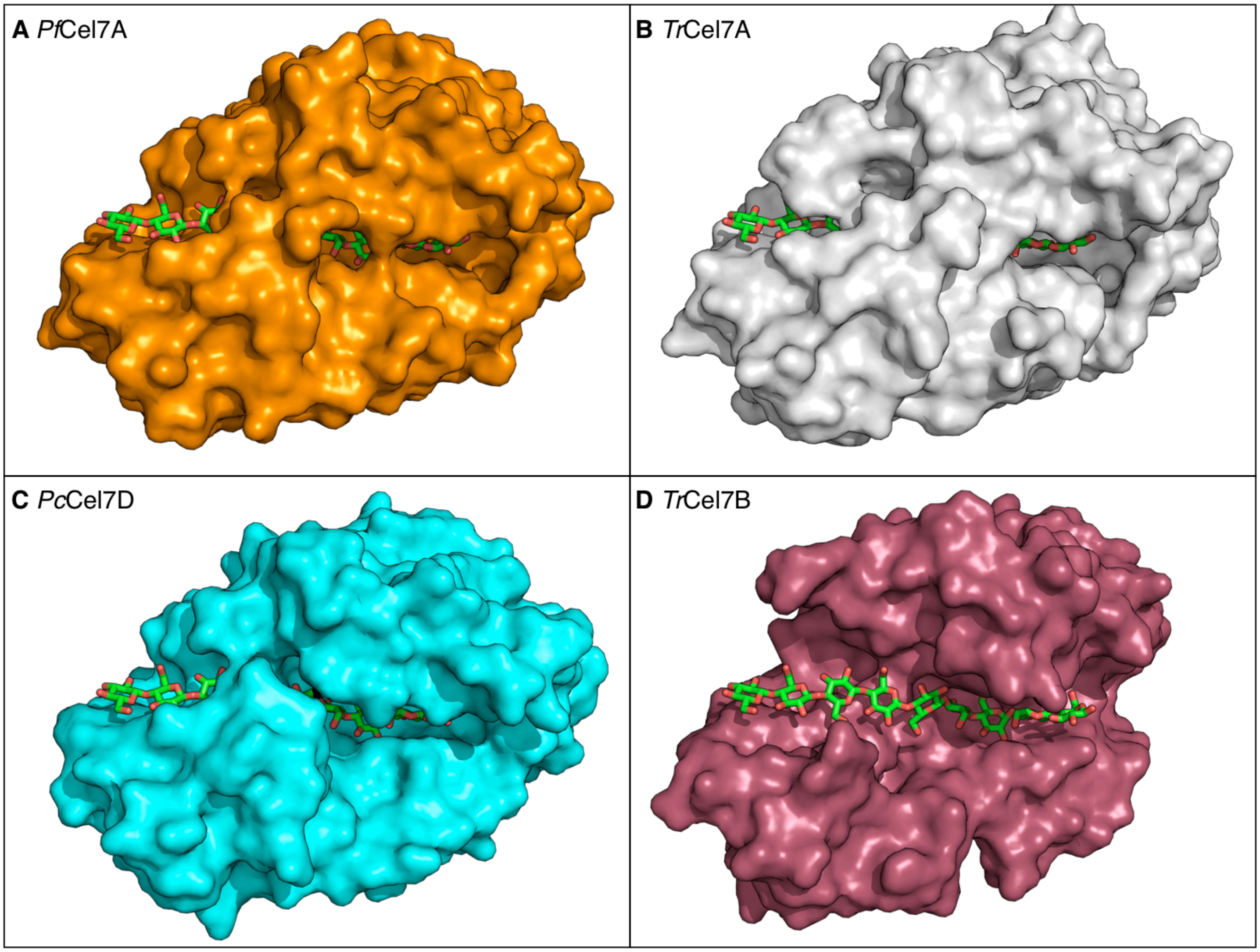

Supplementary Figure 4. Comparison of substrate-binding tunnel enclosure. A) CBH *Pf*Cel7A (PDB code 4XEB). B) CBH *Tr*Cel7A (PDB code 4C4C). C) CBH *Pc*Cel7D. D) Endoglucanase (EG) *Tr*Cel7B (PDB code 1EG1). The degree of openness of the *Pf*Cel7A binding tunnel is intermediate between that of *Tr*Cel7A and *Pc*Cel7D, the latter of which has a significantly shortened B3 loop (aka "exo loop"). EG *Tr*Cel7B is shown for context, demonstrating the large difference between CBHs and EGs. In all four panels, the cellononaose ligand from the *T. reesei* Michaelis complex (PDB code 4C4C) is shown in green "sticks".

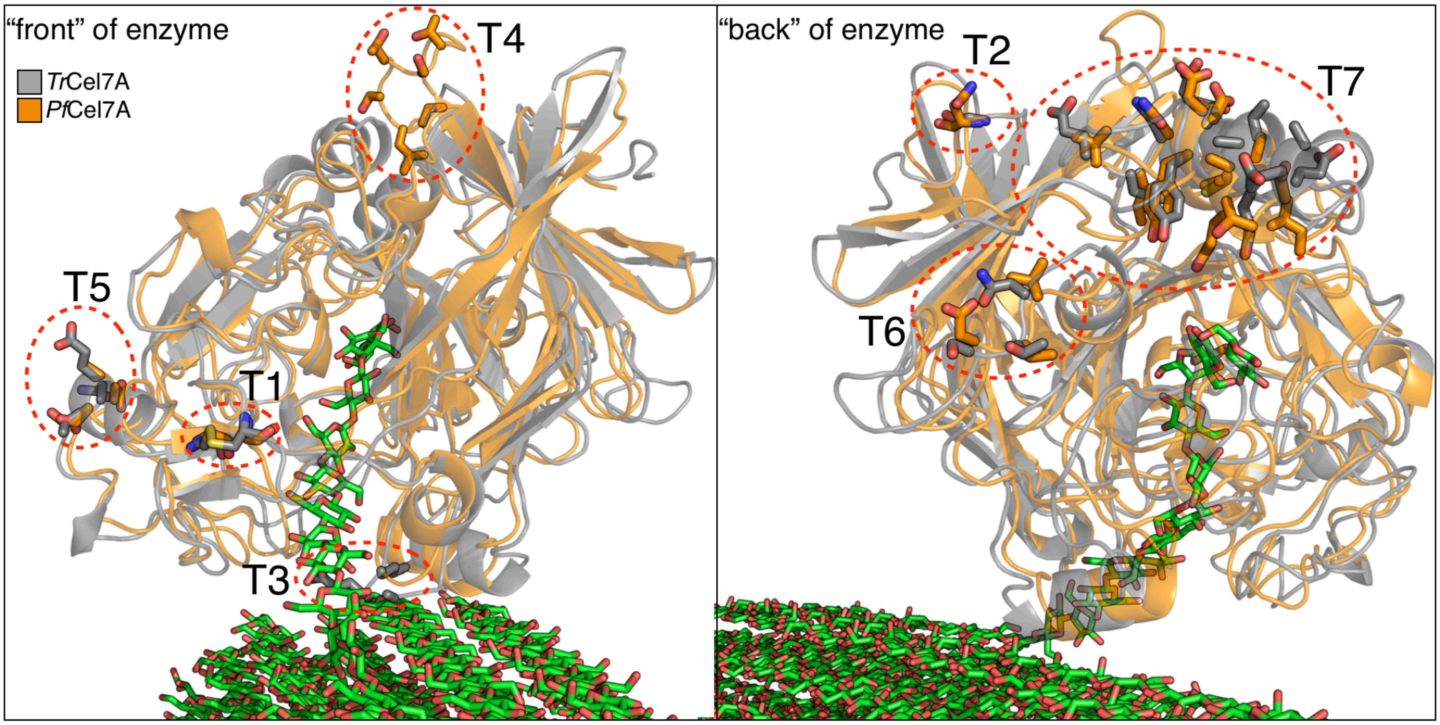

Supplementary Figure 5. Primary regions of structural variation between *Tr*Cel7A and *Pf*Cel7A. In all, seven regions of differentiation were identified, which inspired the construction of a CD mutant library. For each region T1-T7, the motif found in *Pf*Cel7A was introduced into the *Tr*Cel7A parent (thus the "T" in the name). An annotated sequence alignment that highlights each of these regions can be seen in Fig. S6.

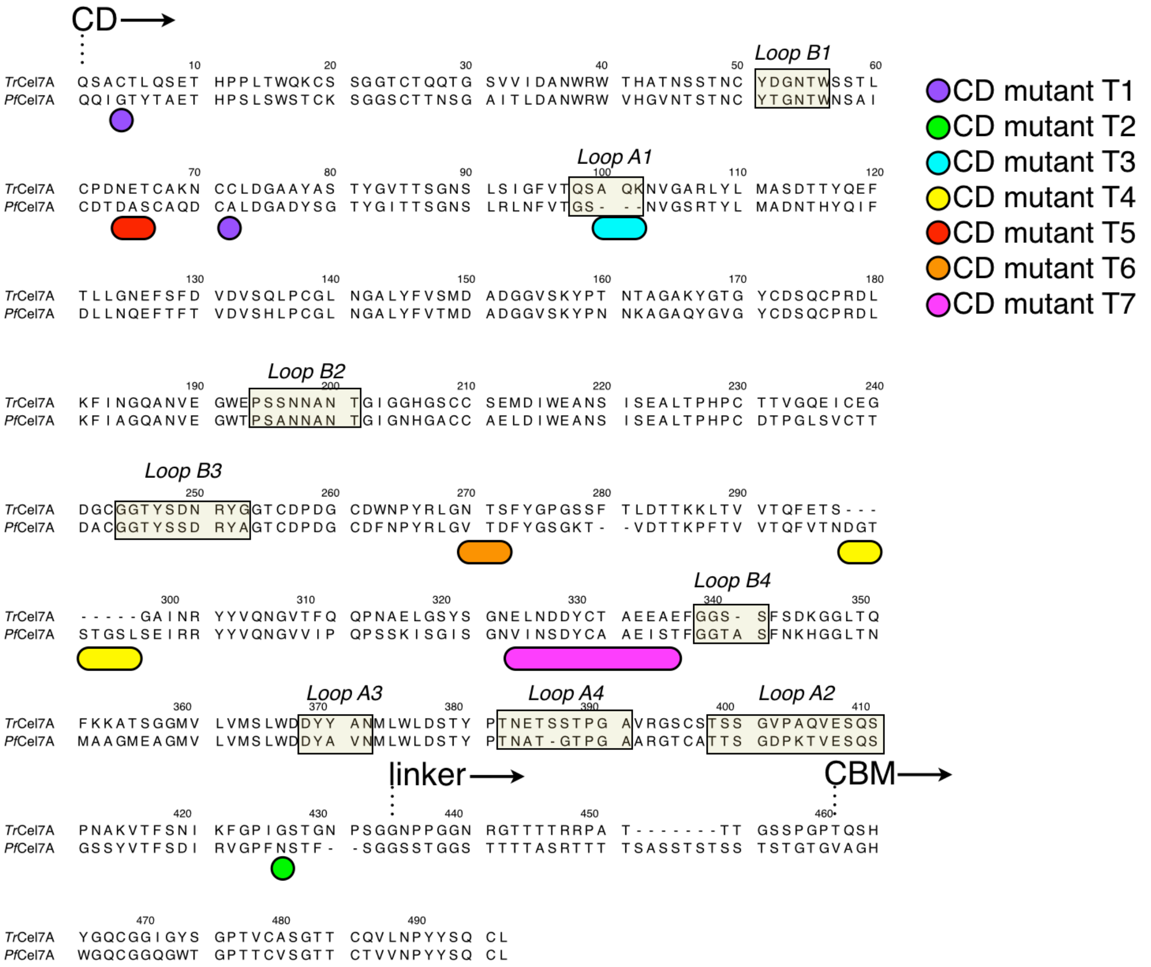

Supplementary Figure 6. Sequence alignment of the full wild-type proteins (CD, linker, and CBM) for *Tr*Cel7A and *Pf*Cel7A. The sequence alignment is annotated with the locations of the loops that form the substrate-binding tunnel (A1-A4, B1-B4). Additionally, the locations of the CD mutant library are shown in various colors as described in the legend at right. The "T" in the names of the CD mutant library refers to the fact that each of these was constructed with the *Tr*Cel7A parent (i.e. the *Pf*Cel7A motif was introduced into the *Tr*Cel7A parent enzyme in each case). The location of each of these regions with respect to the overall CD and the cellulose surface can be seen in Fig. S5.

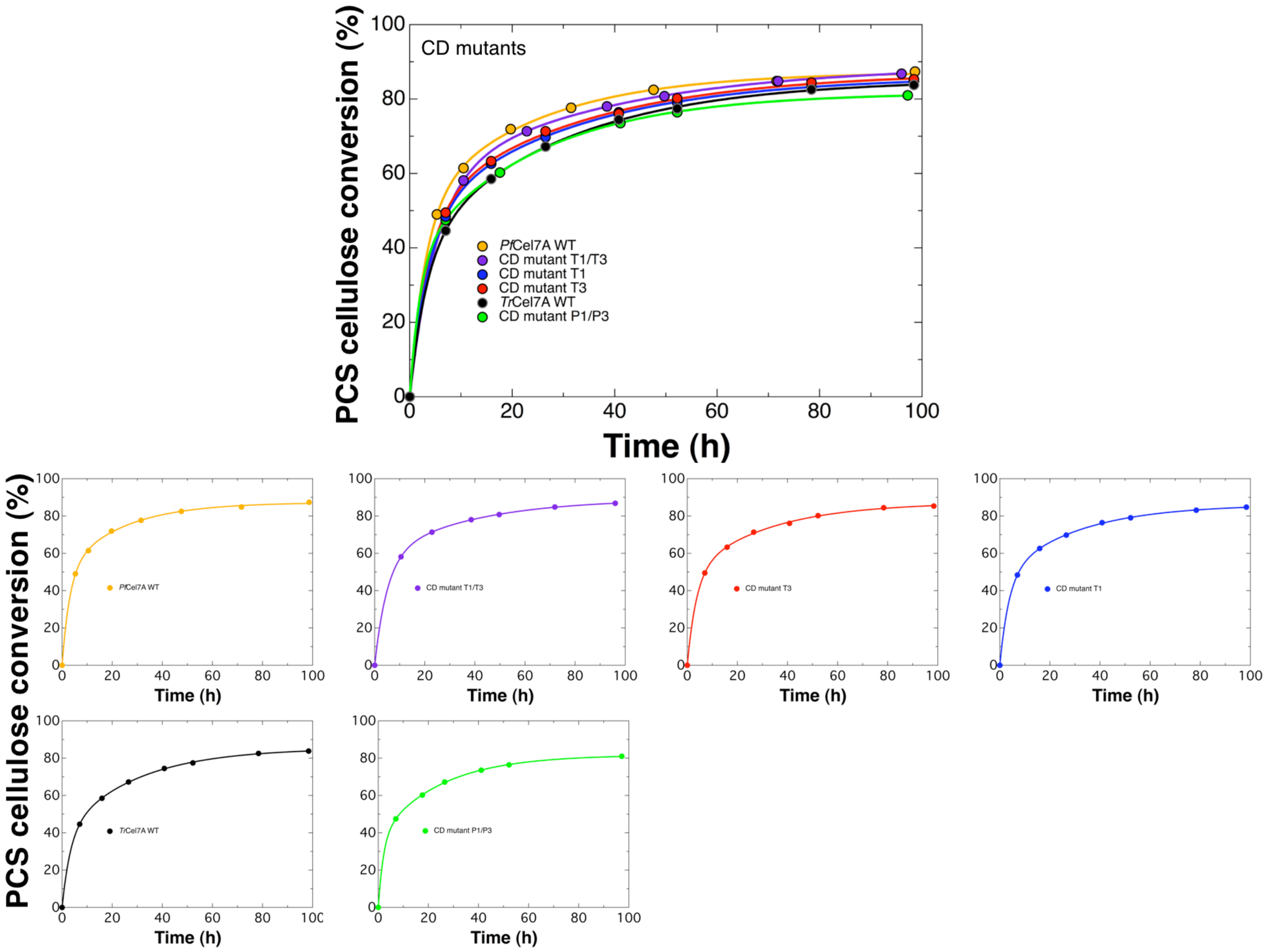

Supplementary Figure 7. Glucan conversion as a function of time on pre-treated corn stover (PCS) for high performing enzymes from the CD mutant library. Data points are shown as circles, and lines represent double-exponential fits to the data. The assays were performed at 40°C and pH 5.0. The time constants for these double exponential fits are represented in the main text Fig. 4. Experiments were performed in triplicate; error bars represent the SEM and are smaller than the data markers.

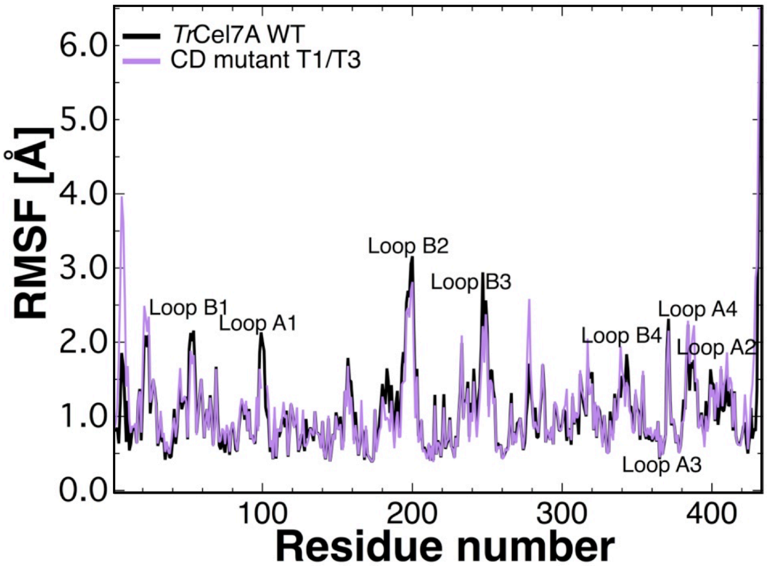

Supplementary Figure 8. Root mean squared fluctuations (RMSF) by residue in MD simulations of CD in solution. Shown are the RMSF profiles for *Tr*Cel7A WT and CD double mutant T1/T3 in solution, with bound cellononaose substrate. The removal of the disulfide bond significantly increases the flexibility of the residues at the binding tunnel entrance, particularly between residues 3-12.

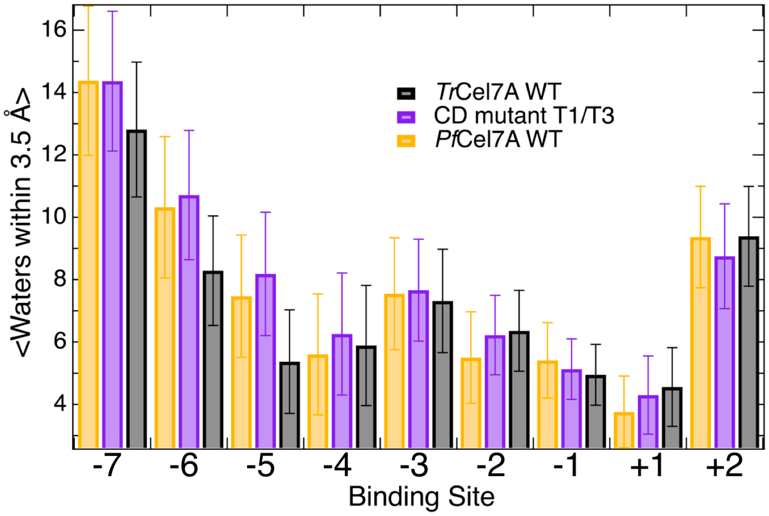

Supplementary Figure 9. Average number of water molecules within 3.5 Å of the glucose moiety in each binding site. The three binding sites nearest to the tunnel entrance (-7 through -5) have, on average, 2-3 more water molecules nearby in the CD mutant T1/T3 (near the levels of *Pf*Cel7A WT) than their counterparts in *Tr*Cel7A WT. Each bar represents the average from a single molecular dynamics trajectory of 1 µs in length for each enzyme. Error bars represent the standard deviation.

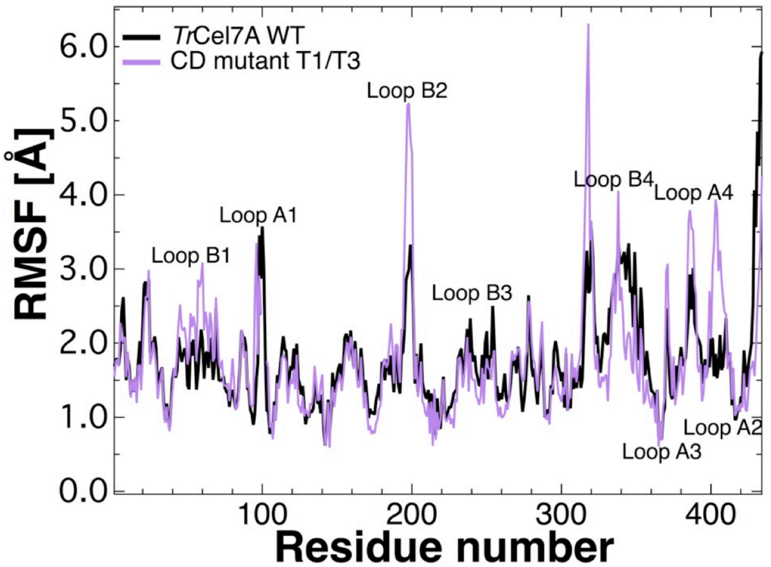

Supplementary Figure 10. Root mean squared fluctuations (RMSF) by residue in MD simulations of full intact proteins (CD, linker, CBM) complexed on the surface of a cellulose microfibril. Shown are the RMSF profiles for *Tr*Cel7A WT and CD double mutant T1/T3. Likely due to the shortening of the A1 loop, a "cascade effect" leads to large scale rearrangements of some loops (A1, A2, and A3, see main text, Fig. 5F) and makes others (B1, B2, A2, A4) more flexible. The trigger for these widespread changes is likely the loss of the stabilizing salt bridge present in the WT between Lys102 (A1 loop) and Glu408 (A2 loop, see main text, Fig. 5E).

Supplementary Table 1. Comparison of Michaelis-Menten kinetic parameters on *p*NPL, Tmax, and pH and temperature optima for *Tr*Cel7A and *Pf*Cel7A

Enzyme	T_{opt} (°C)	pH opt.	T_{max} (°C)	k_{cat} (min^{-1})	K_m (μM)
*Pf*Cel7A	60	4	66	16.34 ± 0.82	2656 ± 692
*Tr*Cel7A	55	4	64	14.26 ± 1.05	2195 ± 880
T1/T3	n.d.	n.d.	63	13.79 ± 0.81	1568 ± 443

Supplementary Table 2. Activity ratio as a function of conversion target.

target conversion (%)	*Pf*Cel7A time to target (h)	*Tr*Cel7A time to target (h)	Activity ratio (*Pf*/*Tr*)
80	37.77	61.77	1.64
75	25.70	42.79	1.66
70	17.98	31.47	1.75
65	12.73	23.42	1.84
60	9.32	17.31	1.86
55	7.11	12.71	1.79
50	5.59	9.43	1.69

Supplementary Methods

Molecular simulations. Molecular dynamics (MD) simulations were performed both in solution and on the surface of a cellulose microfibril.

Solution simulations were performed with WT *Tr*Cel7A, WT *Pf*Cel7A, and CD mutant T1/T3. In each case, a cellononaose ligand was bound in the active site of the CD; this ligand was taken from the WT *Tr*Cel7A Michaelis complex.[3] In all cases, the ligand was docked into the protein via structural alignment of the proteins from the -7 to the +2 sites. The protonation states of the catalytic triad were taken from the proposed retaining mechanism for GH7 enzymes (nucleophile charged, acid/base protonated, and 'helping residue' protonated). Each enzyme was solvated in a box of explicit water molecules of approximate dimensions 80 x 80 x 80 $Å^3$. Sodium ions were added to neutralize the system. The total system size was approximately 52,000 atoms.

MD simulations on the surface a cellulose microfibril were performed for *Tr*Cel7A and CD mutant T1/T3. The construction of the surface simulations followed the protocol of Payne *et al*.[4] The *Tr*Cel7A CD structure was taken from the Protein Data Bank 8CEL with a cellononaose ligand in the tunnel to form the catalytically active complex.[5] The crystal structure of the Michaelis complex (PDB code 4C4C) is now available[3], however the structure was shown to overlay the 8CEL model, so we keep 8CEL as our basis for consistency with the previous simulations. The ten disulfide bridges native to *Tr*Cel7A were constructed and active site residues Asp214 and Glu217 were protonated. The CBM was taken from the NMR structure[6]. In addition to the *N*-glycans attached to the CD utilized previously (at sites Asn45, Asn270, and Asn384), an *N*-glycan is also attached here at Asn64; in all cases, the *N*-glycan is $Man_5GlcNAc_2$. The *O*-glycans on linker and CBM were taken from a detailed mass spectrometry study by Harrison *et al*.[7] and were α-*O*-linked to serine or threonine. Subsequent *O*-linked glycans were bonded as α-1,2 linkages.[8] In addition to the previously simulated *O*-glycans, mannose was also attached at Ser475 on the CBM. The cellulose microfibril is 28 units long with four layers of cellulose chains. The top layer comprises three chains. *Tr*Cel7A is complexed with an edge chain of the top layer on the hydrophobic face of cellulose. Each system contained approximately 116,000 atoms with dimensions of ~160 Å x 80 Å x 80 Å.

The initial simulation of WT *Tr*Cel7A (fully glycosylated) began with the linker unbound and approximately 12 glucose residues of the complexed chain displaced from their crystal structure positions within the microfibril surface. The CD mutant T1/T3 was built from this system after 200 ns of unrestrained molecular dynamics. In this time period, several residues of the cellulose chain anneal back into the surface, leaving only nine glucose residues outside of the microfibril. The linker domain also binds to the cellulose surface within 200 ns. Thus, the mutant simulation begins with the linker bound and the complexed cellulose chain in a relaxed configuration. From this point, both systems were simulated for an additional 1 μs.

For all systems, the CHARMM force field with CMAP was used to describe the proteins [9], the carbohydrates were described with the C35 force field [10, 11], and water was described with the TIP3P model.[12] All MD simulations utilized explicit solvent with sodium ions added to achieve charge neutrality. The systems were built and minimized in CHARMM[13], performing a stepwise protocol of minimization in which restraints on protein, cellulose chain, and entire cellulose surface (where applicable, excepting the bottom layer) are gradually released. Systems were density equilibrated for 1 ns at a constant pressure of 1 atmosphere and constant temperature of 300 K (Nosé-Hoover barostat and thermostat); subsequent production runs were performed with constant volume and temperature (300 K) in NAMD.[14] The SHAKE algorithm was utilized to fix all bonded hydrogen distances.[15] The timestep was 2 fs. Nonbonded cutoff distance of 10 Å was utilized, with a switching distance of 9 Å, and a nonbonded pair list distance of 13 Å. The Particle Mesh Ewald method was used to describe the long-range electrostatics[16] with a sixth order b-spline, a Gaussian distribution with a width of 0.312 Å, and 1 Å grid spacing. The velocity Verlet multiple timestepping integration scheme was used evaluating the full nonbonded interactions every 2 timesteps, with full electrostatics interactions every 4 timesteps, and 20 timesteps between atom reassignments. For the surface systems, all minimizations and simulations were performed with the bottom layer of the cellulose slab harmonically restrained with a force constant of 5 $kcal \bullet mole^{-1} \bullet Å^{-2}$ on the glucopyranose ring atoms.

Supplementary References

1. Murshudov, G. N. et al. REFMAC5 for the refinement of macromolecular crystal structures. *Acta Crystallogr. Sect. D-Biol. Crystallogr.* **67**, 355-367 (2011).

2. The PyMOL Molecular Graphics System, Version 2.0 Schrödinger, LLC.

3. Knott, B. C. et al. The mechanism of cellulose hydrolysis by a two-step, retaining cellobiohydrolase elucidated by structural and transition path sampling studies. *J. Am. Chem. Soc.* **136**, 321–329 (2014).

4. Payne, C. M. et al. Glycosylated linkers in multi-modular lignocellulose degrading enzymes dynamically bind to cellulose. *Proc. Natl. Acad. Sci. USA* **110**, 14646-14651 (2013).

5. Divne, C., Ståhlberg, J., Teeri, T. T. & Jones, T. A. High-resolution crystal structures reveal how a cellulose chain is bound in the 50 Å long tunnel of cellobiohydrolase I from *Trichoderma reesei*. *J. Mol. Biol.* **275**, 309-325 (1998).

6. Kraulis, J. et al. Determination of the three-dimensional solution structure of the C-terminal domain of cellobiohydrolase I from *Trichoderma reesei.* A study using nuclear magnetic resonance and hybrid distance geometry-dynamical simulated annealing. *Biochemistry-US* **28**, 7241-7257 (1989).

7. Harrison, M. J. et al. Modified glycosylation of cellobiohydrolase I from a high cellulase-producing mutant strain of *Trichoderma reesei*. *Eur. J. Biochem.* **256**, 119-127 (1998).

8. Deshpande, N., Wilkins, M. R., Packer, N. & Nevalainen, H. Protein glycosylation pathways in filamentous fungi. *Glycobiology* **18**, 626-637 (2008).

9. Mackerell, A. D., Feig, M. & Brooks, C. L. Extending the treatment of backbone energetics in protein force fields: Limitations of gas-phase quantum mechanics in reproducing protein conformational distributions in molecular dynamics simulations. *J. Comput. Chem.* **25**, 1400-1415 (2004).

10. Guvench, O., Hatcher, E., Venable, R. M., Pastor, R. W. & MacKerell, A. D. CHARMM additive all-atom force field for glycosidic linkages between hexopyranoses. *J. Chem. Theory Comput.* **5**, 2353-2370 (2009).

11. Guvench, O. et al. CHARMM additive all-atom force field for carbohydrate derivatives and its utility in polysaccharide and carbohydrate-protein modeling. *J. Chem. Theory Comput.* **7**, 3162-3180 (2011).

12. Jorgensen, W. L., Chandrasekhar, J. & Madura, J. D. Comparison of simple potential functions for simulating liquid water. *J. Chem. Phys.* **79**, 926-935 (1983).

13. Brooks, B. R. et al. CHARMM: the biomolecular simulation program. *J. Comput. Chem.* **30**, 1545-1614 (2009).

14. Phillips, J. C. et al. Scalable molecular dynamics with NAMD. *J. Comput. Chem.* **26**, 1781-1802 (2005).

15. Ryckaert, J., Ciccotti, G. & Berendsen, H. Numerical integration of the Cartesian equations of motion of a system with constraints: molecular dynamics of n-alkanes. *J Comput Phys* **23**, 327-341 (1977).

16. Essmann, U. et al. A smooth particle mesh Ewald method. *J. Chem. Phys.* **103**, 8577-8593 (1995).
